# Predictive Values of the New Sarcopenia Index by the Foundation for the National Institutes of Health Sarcopenia Project for Mortality among Older Korean Adults

**DOI:** 10.1371/journal.pone.0166344

**Published:** 2016-11-10

**Authors:** Joon Ho Moon, Kyoung Min Kim, Jung Hee Kim, Jae Hoon Moon, Sung Hee Choi, Soo Lim, Jae-Young Lim, Ki Woong Kim, Kyong Soo Park, Hak Chul Jang

**Affiliations:** 1 Department of Internal Medicine, Seoul National University College of Medicine, Seoul, Korea; 2 Department of Internal Medicine, Seoul National University Hospital, Seoul, Korea; 3 Department of Internal Medicine, Seoul National University Bundang Hospital, Seongnam, Korea; 4 Department of Rehabilitation Medicine, Seoul National University Bundang Hospital, Seongnam, Korea; 5 Department of Neuropsychiatry, Seoul National University Bundang Hospital, Seongnam, Korea; West Virginia University School of Medicine, UNITED STATES

## Abstract

**Objective:**

We evaluated the Foundation for the National Institutes of Health (FNIH) Sarcopenia Project’s recommended criteria for sarcopenia’s association with mortality among older Korean adults.

**Methods:**

We conducted a community-based prospective cohort study which included 560 (285 men and 275 women) older Korean adults aged ≥65 years. Muscle mass (appendicular skeletal muscle mass-to-body mass index ratio (ASM/BMI)), handgrip strength, and walking velocity were evaluated in association with all-cause mortality during 6-year follow-up. Both the lowest quintile for each parameter (ethnic-specific cutoff) and FNIH-recommended values were used as cutoffs.

**Results:**

Forty men (14.0%) and 21 women (7.6%) died during 6-year follow-up. The deceased subjects were older and had lower ASM, handgrip strength, and walking velocity. Sarcopenia defined by both low lean mass and weakness had a 4.13 (95% CI, 1.69–10.11) times higher risk of death, and sarcopenia defined by a combination of low lean mass, weakness, and slowness had a 9.56 (3.16–28.90) times higher risk of death after adjusting for covariates in men. However, these significant associations were not observed in women. In terms of cutoffs of each parameter, using the lowest quintile showed better predictive values in mortality than using the FNIH-recommended values. Moreover, new muscle mass index, ASM/BMI, provided better prognostic values than ASM/height^2^ in all associations.

**Conclusions:**

New sarcopenia definition by FNIH was better able to predict 6-year mortality among Korean men. Moreover, ethnic-specific cutoffs, the lowest quintile for each parameter, predicted the higher risk of mortality than the FNIH-recommended values.

## Introduction

Populations are rapidly aging and this has become a global health burden [1]. Aging is accompanied by body composition changes, and Rosenberg first proposed the term ‘sarcopenia’ to describe age-related pathologic loss of skeletal muscle mass [2]. Skeletal muscles play a crucial role in humans, and therefore sarcopenia has resulted in various adverse health outcomes including fracture, fall, disability, and mortality [3, 4].

Sarcopenia was initially defined as relative appendicular skeletal muscle mass (ASM) less than 2 standard deviations below the mean values of healthy young adults or the lowest quintile of study populations [4, 5]. ASM divided by height squared (ASM/ht^2^) was first proposed by Baumgartner as a representative method to estimate relative ASM [4]. Recent studies, however, indicated that decline in muscle strength or physical performance may be more important in health outcomes related to sarcopenia [6]. Based on this, the criteria combining ‘reduced muscle mass with either muscle weakness or poor physical performance’ has been proposed by study groups including the European Working Group for Sarcopenia in Older People and the Asian Working Group for Sarcopenia (AWGS) [7, 8]. These criteria demonstrated better associations with various outcomes than the definitions driven only by muscle mass [9, 10]. A number of working groups have suggested different criteria for sarcopenia, but each has different clinical implications [11] and no consensus has been achieved for ‘gold standard’ criteria for sarcopenia. The Foundation for the National Institutes of Health (FNIH) Biomarkers Consortium initiated ‘The FNIH Sarcopenia Project’ to derive, using the best available data, criteria for clinically relevant low lean mass and weakness that correspond to incident mobility impairment [12]. A total of 26,625 participants from nine large community-based cohorts were pooled for the analysis, and the new FNIH criteria were proposed in 2014, the cutoff values for which are presented in S1 Table [12]. In particular, a new muscle mass index of ‘ASM divided by body mass index (BMI)’ instead of ASM/ht^2^ or ASM/weight was suggested by the FNIH. Different measures of lean mass (*i*.*e*., ASM and leg lean mass) standardized to various body size measures (*i*.*e*., height^2^, weight, BMI, total body fat) were tested using the classification and regression tree (CART) analysis, and ASM/BMI was selected as a discriminator of clinical weakness [13]. There have been attempts to clarify the clinical implications of this new FNIH criteria [10, 11, 14], but still there is sparse data about associations between the new FNIH criteria and functional outcomes.

In the FNIH Sarcopenia Project, mobility limitation was the primary outcome for deriving cutoffs for low lean mass and weakness. On the other hand, mortality, another clinically important geriatric outcome, is often an outcome in studies of frailty [15, 16]. In recent studies, mortality in association with measures of sarcopenia was also evaluated in community-dwelling older adults, broadening the application of the definitions of sarcopenia [3, 17, 18]. In this context, we investigated the association between the FNIH-proposed new sarcopenia definition and 6-year mortality, using the data from the prospective longitudinal cohort consisting of community-dwelling older adults in Korea. In the analysis, we used both the lowest quintile for each sarcopenia parameter as ethnic-specific cutoff and FNIH-recommended cutoff values to compare the predictability of mortality among Asian population.

## Materials and Methods

### Subjects

This study was conducted as a part of the Korean Longitudinal Study on Health and Aging (KLoSHA), a community-based cohort study initiated to investigate health, aging, and geriatric diseases of Korean older adults [19]. In August 2005, residents in Seongnam, South Korea aged 65 years or older were invited by letter or telephone to participate. A total of 439 men and 561 women agreed to participate in the study, and the baseline evaluation was conducted from September 2005 to September 2006. From this source population, 560 subjects (285 men and 275 women) who performed all muscle-related measures were included in the analysis. All participants were fully informed regarding the study participation, and written informed consent was obtained from every subjects. The study was conducted following the Declaration of Helsinki, and approved by the Institutional Review Board of Seoul National University Bundang Hospital (IRB Number: B-0706-046-012, B-1506-304-112).

### Muscle mass

Body composition was assessed by dual energy x-ray absorptiometry (Lunar Corporation, Madison, WI, USA). ASM was calculated as the sum of the lean mass in both arms and legs.

### Handgrip strength

Handgrip strength was measured to evaluate muscle strength (Jamar Hydraulic Dynamometer, Sammons Preston, Bolingbrook, IL, USA). In a sitting position with shoulder adducted, elbow flexed at 90°, and forearm in the neutral position, participants were asked to grasp the dynamometer at their maximal power [20]. Two serial attempts by the dominant hand in the neutral position were measured with a one-minute rest, and the values were averaged for analysis.

### Walking velocity

Walking velocity was used to assess participants’ physical performance. Subjects were asked to walk across a 4-meter distance at comfortable speed on a flat level with no obstructions [21].

### Definition of sarcopenia

We used both the lowest quintile of study subjects and FNIH-recommended values for cutoffs to define sarcopenia [12]. AWGS criteria [7] were used to compare the predictability with the FNIH criteria. Cutoff points are presented in S1 Table. ‘Sarcopenia_mass/strength_’ was defined as having both weakness and low lean mass, and ‘Sarcopenia_mass/strength/performance_’ as having low lean mass, weakness, and slowness [22].

### Mortality

Mortality data were collected by an annual telephone call, and were confirmed by the data retrieved from the National Death Registry. Follow-up duration was from the baseline evaluation until the date of death or the last follow-up on December, 2011.

### Functional performance measures

Activities of daily living (ADL), instrumental activities of daily living (IADL), and the short physical performance battery (SPPB) [19, 23] were assessed at baseline and at the 5-year follow-up evaluation. ADL and IADL were evaluated by questionnaire. The SPPB evaluates lower-extremity performance and comprises three components: standing balance, chair stand, and gait speed. Each component is scored from 1 to 4, and the maximum total score is 12 [21].

### Anthropometric and biochemical measurements

Anthropometric measures including height, weight, and blood pressure were measured by standardized procedures. Blood was drawn after a fast of more than eight hours. Serum glucose was measured using the glucose-oxidase method (YSI 2300 STAT Glucose Analyzer, YSI Inc., Yellow Springs, OH, USA). Total cholesterol, creatinine, and alanine transaminase (ALT) were enzymatically measured (Hitachi 747, Hitachi Ltd., Tokyo, Japan). TSH and free T4 concentrations were measured by immunoradiometric assays (TSH: CIS Bio International, Codolet, France; free T4: LIAISON, DiaSorin S.p.A., Saluggia, Italy).

### Covariates

Smoking history, alcohol consumption, and physical activity data were acquired by questionnaire. Exercise was defined as performing ≥150 minutes of moderate-intensity or ≥75 minutes of vigorous-intensity physical activities per week [24]. Comorbidities including diabetes, hypertension, cardiovascular disease, stroke, and osteoarthritis were acquired based on self-report, clinical diagnosis, and medication use. The cumulative illness rating scale (CIRS) was used to assess the general medical condition of older adults [25].

### Statistical analysis

Baseline characteristics were compared by their survival status in each gender. Differences among groups were evaluated using Student’s *t* test for continuous variables and χ^2^ test for categorical variables. Data are presented as the mean ± standard deviation (SD) or number of subjects (%). Mortality by different muscle-related parameters was assessed, and the risk is presented as relative risk and 95% confidence interval (CI). Then, we used the Cox proportional hazard model for survival analysis. For the primary analysis (Model 1), unadjusted hazard ratio (HR) and 95% CI for mortality were estimated. In Model 2, age, BMI, systolic blood pressure, fasting glucose, total cholesterol, creatinine, ALT, free T4, and CIRS were used as covariates in the Cox model. In Model 3, smoking history, alcohol consumption, and physical activity were used as covariates in addition to Model 2. Statistical analyses were performed with SPSS version 20.0 (IBM Co., Armonk, NY, U.S.A.). Differences were considered significant when *P* < .05.

## Results

A total of 560 subjects (285 men and 275 women) were included in the final analyses. Mean age of the study subjects was 73.8 ± 7.4 years old and mean BMI was 24.4 ± 3.2 kg/m^2^. During the 6-year follow-up, a total of 61 subjects (40 men and 21 women) died. Subjects’ baseline characteristics are summarized by their survival status at the 6-year follow-up evaluation in Table 1. In both genders, subjects who died were older and had lower ASM, handgrip strength, and walking velocity.

**Table 1:** Baseline Characteristics According to Survival in Men and Women.

N	Men			Women		
	Alive	Death	*P*-value	Alive	Death	*P*-value
	245 (86.0%)	40 (14.0%)		254 (92.4%)	21 (7.6%)	
Age (year)	73.3±7.2	80.4±8.9	<0.001*	72.8±6.71	78.1±8.51	0.001*
BMI (kg/m^2^)	24.1±3.1	23.8±4.0	0.626	24.7±3.1	23.7±3.4	0.133
Smoking, n (%)						
Non/Ex-/Current smoker	57/140/48 (23.3/57.1/19.6%)	11/18/11 (27.5/45.0/27.5%)	0.331	237/7/10 (93.8/2.5/3.6%)	21/0/0 (100/0/0%)	0.473
Current drinker, n (%)	122 (49.8%)	12 (30.0%)	0.020	20 (7.9%)	0 (0.0%)	0.182
Regular exercise, n (%)	179 (73.1%)	26 (65.0%)	0.293	111 (43.7%)	5 (23.8%)	0.076
Stroke history, n (%)	23 (9.5%)	7 (17.5%)	0.131	32 (12.8%)	3 (14.3%)	0.845
Hypertension, n (%)	165 (67.3%)	25 (62.5%)	0.547	174 (68.5%)	14 (66.7%)	0.862
Type 2 Diabetes, n (%)	101 (41.2%)	22 (55.0%)	0.103	99 (39.0%)	3 (14.3%)	0.024*
CIRS	3.83±2.55	3.90±2.42	0.862	3.90±2.47	4.25±2.67	0.540
Systolic BP (mmHg)	133.7±17.6	134.8±21.7	0.728	133.1±21.1	135.2±18.6	0.659
Diastolic BP (mmHg)	83.8±10.9	83.3±12.7	0.758	83.6±11.7	83.8±10.2	0.947
Fasting glucose (mg/dL)	114.7±27.6	115.3±26.0	0.904	108.7±24.9	103.1±14.5	0.308
Total cholesterol (mg/dL)	195.4±38.8	183.4±30.7	0.064	209.7±36.4	212.9±26.5	0.697
Creatinine (mg/dL)	1.22±0.23	1.29±0.29	0.111	0.99±0.17	1.08±0.29	0.057
ALT (mg/dL)	24.3±13.1	19.4±10.7	0.025*	23.6±23.4	22.1±13.9	0.777
TSH (mIU/L)	3.24±3.56	3.62±6.13	0.219	3.19±2.77	4.10±3.60	0.033*
free T4 (ng/dL)	1.27±0.37	1.27±0.34	0.580	1.19±0.39	1.18±0.34	0.160
***Sarcopenia parameters***						
ASM (kg)	20.24±2.59	18.99±3.31	0.007*	13.66±1.84	12.74±1.48	0.027*
ASM/ht^2^ (kg/m^2^)	7.42±0.82	7.08±0.97	0.018*	5.97±0.66	5.82±0.50	0.308
ASM/BMI	0.85±0.10	0.81±0.13	0.029*	0.56±0.07	0.54±0.05	0.381
Handgrip strength (kg)	27.17±8.38	21.01±7.88	<0.001*	13.99±5.49	10.48±5.81	0.005*
Walking velocity (m/s)	0.93±0.30	0.72±0.29	<0.001*	0.77±0.30	0.57±0.30	0.003*

Values are presented as mean ± standard deviation for continuous variable or number (%) for dichotomous variable. ASM, appendicular skeletal muscle mass; CIRS, the cumulative illness rating scale; ht^2^, height squared.

*P < 0.05.

Regarding muscle-related parameters according to survival status, all quantitative muscle mass indices including ASM, ASM/ht^2^, and ASM/BMI were significantly lower in deceased men than in surviving men. However in women, the only significant difference between deceased and survived subjects was on ASM. Handgrip strength and walking velocity were significantly lower in deceased subjects in both genders.

First, we compared baseline functional performance measures (*i*.*e*., ADL, IADL, and SPPB) with sarcopenia defined by the FNIH criteria. Subjects with Sarcopenia_mass/strength_ (20%) or Sarcopenia_mass/strength/performance_ (20%) had higher IADL and lower SPPB than those without sarcopenia, but the difference in ADL did not reach statistical significance (S2 Table). However, in longitudinal analyses, there were no significant relationships between changes in these three functional performance measures and sarcopenic status (S3 Table).

Next, we evaluated relative risk for 6-year mortality of respective parameters including muscle mass indices, handgrip strength, physical performance, and composite of those parameters (Table 2). In men, relative risk for mortality was significantly increased for low ASM/BMI, handgrip strength, and walking velocity. The two composite indices, Sarcopenia_mass/strength_ and Sarcopenia_mass/strength/performance_, provided higher predictive values for mortality than individual parameters; in men, Sarcopenia_mass/strength_ (20%) had a 4.88-fold risk of mortality, and Sarcopenia_mass/strength/performance_ (20%) had a 7.96-fold risk. Men with sarcopenia defined by the AWGS criteria had a 2.63-times increased risk of mortality compared with men without sarcopenia. In women, relative risk for mortality was significantly increased for low handgrip strength and walking velocity, but low muscle mass (ASM/BMI) was not associated with mortality. Sarcopenia_mass/strength_ (20%) had a 3.10-fold increased risk of mortality, but Sarcopenia_mass/strength/performance_ (20%) did not show significantly increased risk. Sarcopenia defined by the AWGS criteria did not predict mortality in women. In a subgroup analysis by age (65–74 years and >75 years), both subgroups of men showed a significant association between sarcopenia and mortality, whereas in women, no significant association was found except for with Sarcopenia_mass/strength/performance_ (20%) in women aged 65–74 years (S4 Table).

**Table 2:** Six-year Mortality and Its Relative Risk by Sarcopenia Parameters.

	Alive	Death	Relative risk (95% CI)	*P-*value
**Men (N = 285)**	N = 245	N = 40		
***Muscle mass***				
By ASM/ht^2^ (20%)	42 (17.1%)	15 (37.5%)	2.40 (1.36–4.25)	0.003*
By ASM/BMI (20%)	41 (16.1%)	16 (28.1%)	2.67 (1.52–4.68)	0.001*
By ASM/BMI (FNIH)	72 (29.4%)	20 (50.0%)	2.10 (1.19–3.70)	0.010*
***Muscle strength***				
By handgrip (20%)	42 (17.1%)	19 (47.5%)	3.32 (1.91–5.77)	<0.001*
By handgrip (FNIH)	110 (44.9%)	31 (77.5%)	3.51 (1.74–7.12)	<0.001*
***Physical performance***				
By walking velocity (20%)	41 (16.7%)	18 (45.0%)	3.13 (1.80–5.45)	<0.001*
By walking velocity (FNIH)	117 (47.8%)	31 (77.5%)	3.19 (1.58–6.45)	<0.001*
***In combination***				
By Sarcopenia_mass/strength_ (20%)	11 (4.5%)	12 (30.0%)	4.88 (2.89–8.25)	<0.001*
By Sarcopenia_mass/strength_ (FNIH)	39 (15.9%)	17 (42.5%)	3.02 (1.74–5.26)	<0.001*
By Sarcopenia_mass/strength/performance_ (20%)	1 (0.4%)	9 (22.5%)	7.96 (5.40–11.80)	<0.001*
By Sarcopenia_mass/strength/performance_ (FNIH)	24 (9.8%)	16 (40.0%)	4.08 (2.39–6.99)	<0.001*
By Sarcopenia (AWGS)	54 (22.0%)	19 (47.5%)	2.63 (1.50–4.60)	<0.001*
**Women (N = 275)**	N = 254	N = 21		
***Muscle mass***				
By ASM/ht^2^ (20%)	51 (20.1%)	4 (19.0%)	0.94 (0.33–2.69)	0.910
By ASM/BMI (20%)	51 (20.1%)	4 (19.0%)	0.94 (0.33–2.69)	0.910
By ASM/BMI (FNIH)	71 (28.0%)	5 (23.8%)	0.82 (0.31–2.16)	0.683
***Muscle strength***				
By handgrip (20%)	43 (16.9%)	12 (57.1%)	5.33 (2.37–12.02)	0.001*
By handgrip (FNIH)	161 (63.9%)	18 (85.7%)	3.22 (0.97–10.65)	0.055
***Physical performance***				
By walking velocity (20%)	47 (18.7%)	8 (38.1%)	4.45 (1.69–11.73)	0.033*
By walking velocity (FNIH)	177 (70.2%)	19 (90.5%)	1.84 (0.91–16.06)	0.048*
***In combination***				
By Sarcopenia_mass/strength_ (20%)	11 (4.3%)	3 (14.3%)	3.10 (1.04–9.31)	0.046*
By Sarcopenia_mass/strength_ (FNIH)	52 (20.5%)	5 (23.8%)	1.20 (0.46–3.12)	0.716
By Sarcopenia_mass/strength/performance_ (20%)	6 (2.4%)	2 (9.5%)	3.51 (0.98–12.59)	0.061
By Sarcopenia_mass/strength/performance_ (FNIH)	43 (16.9%)	5 (23.8%)	1.48 (0.57–3.84)	0.423
By Sarcopenia (AWGS)	41 (16.1%)	3 (14.3%)	0.88 (0.27–2.84)	0.824

Data presented are number of subjects with sarcopenia. Numbers in parentheses (%) are proportion of subjects with sarcopenia among alive or deceased subjects. ASM, appendicular skeletal muscle mass; ht^2^, height squared; (20%), Lowest quintile for each variable in each gender were used to classify sarcopenia; (FNIH), Cutoff values recommended by FNIH were used to classify sarcopenia; Sarcopenia_mass/strength_ was defined as low muscle mass with low muscle strength; Sarcopenia_mass/strength/performance_ was defined as slowness with both low muscle mass and strength.

*P < 0.05.

**Fig 1:**
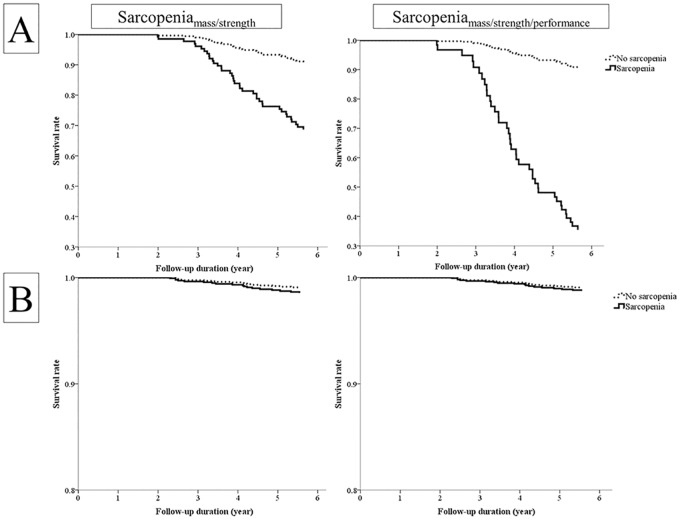
Survival Curve by Sarcopenia in Each Gender. Cumulative survival according to Sarcopenia_mass/strength_and Sarcopenia_mass/strength/performance_ is presented by the Cox proportional hazard model in men (A) and women (B). The model was adjusted for age, BMI, smoking, alcohol, exercise, systolic blood pressure, fasting glucose, total cholesterol, creatinine, alanine transaminase, free T4, and the cumulative illness rating scale.

We further investigated the risk of mortality by each sarcopenia parameter using the Cox proportional hazard model. HRs for 6-year mortality by sarcopenia parameters are shown in Table 3. In the unadjusted model, all measures, including muscle mass, muscle strength, and physical performance, predicted the risk for mortality in men. The composite parameters had higher HRs than individual parameters. Moreover, indices using cutoffs at the lowest quintile showed better prognostic values than those using FNIH cutoff values in all parameters except handgrip strength. After adjusting for conventional risk factors (Models 2 and 3), low lean mass, only those defined by ASM/BMI maintained statistical significance, but ASM/ht^2^ did not show significant associations. Slowness was associated with increased mortality after adjustments, whereas muscle strength indices lost statistical significance. Using the lowest quintile, Sarcopenia_mass/strength_ and Sarcopenia_mass/strength/performance_ predicted increased mortality by 4.13 and 9.56 times respectively, and these values were much higher than those of muscle mass or physical performance alone. However, both composite parameters had decreased predictability (HR 2.48 and 3.83, respectively) when they were defined by FNIH cutoffs. Sarcopenia defined by the AWGS criteria did not have a significant association with mortality after adjusting for covariates. In women, muscle mass indices were not associated with increased risk of mortality in any of the models (Table 3). Low muscle strength, slowness, and composite parameters showed increased risk of mortality in the unadjusted model when defined by the lowest quintile. After adjusting for covariates, however, no sarcopenia parameters including the FNIH criteria or the AWGS criteria, except for handgrip strength, remained to predict mortality. Survival curves by Sarcopenia_mass/strength_ and Sarcopenia_mass/strength/performance_ are presented with Cox proportional hazard model in Fig 1. In men, the difference in survival rate was pronounced after 3 years of follow-up and this trend was exaggerated in longer follow-up, but survival curves did not differ significantly by sarcopenia status in women.

**Table 3:** Hazard Ratios for 6-year Mortality by Sarcopenia Parameters.

	Model 1			Model 2			Model 3			
	HR	95% CI	*P*-value	HR	95% CI	*P*-value	HR		95% CI	*P*-value
**Men (N = 285)**										
***Muscle mass***										
By ASM/ht^2^ (20%)	2.557	1.348–4.851	0.004*	1.752	0.856–3.584	0.125	1.528		0.721–3.235	0.268
By ASM/BMI (20%)	3.047	1.618–5.738	0.001*	3.563	1.692–7.506	0.001*	3.015		1.404–6.472	0.005*
By ASM/BMI (FNIH)	2.306	1.241–4.287	0.008*	2.643	1.317–5.305	0.006*	2.234		1.090–4.582	0.028*
***Muscle strength***										
By handgrip (20%)	3.727	2.003–6.935	<0.001*	1.929	0.913–4.075	0.085	1.688		0.796–3.582	0.374
By handgrip (FNIH)	3.821	1.819–8.027	<0.001*	2.199	0.894–5.407	0.086	1.464		0.701–4.486	0.226
***Physical performance***										
By walking velocity (20%)	3.592	1.926–6.700	<0.001*	2.427	1.232–4.778	0.010*	2.403		1.209–4.777	0.012*
By walking velocity (FNIH)	3.472	1.653–7.294	<0.001*	2.593	1.204–5.583	0.015*	2.400		1.107–5.204	0.027*
***In combination***										
By Sarcopenia_mass/strength_ (20%)	6.988	3.544–13.779	<0.001*	5.095	2.105–12.336	<0.001*	4.128		1.686–10.110	0.002*
By Sarcopenia_mass/strength_ (FNIH)	3.557	1.899–6.660	<0.001*	3.033	1.451–6.343	0.003*	2.477		1.161–5.285	0.019*
By Sarcopenia_mass/strength/performance_ (20%)	19.111	8.902–41.026	<0.001*	11.423	3.992–32.690	<0.001*	9.560		3.162–28.903	<0.001*
By Sarcopenia_mass/strength/performance_ (FNIH)	5.124	2.719–9.657	<0.001*	4.447	2.116–9.342	<0.001*	3.832		1.787–8.219	0.001*
By Sarcopenia (AWGS)	2.833	1.523–5.270	0.001*	1.834	0.887–3.789	0.101	1.451		0.686–3.066	0.330
**Women (N = 275)**										
***Muscle mass***										
By ASM/ht^2^ (20%)	0.942	0.317–2.801	0.915	1.082	0.339–3.449	0.894	0.959	0.301–3.056		0.943
By ASM/BMI (20%)	0.947	0.319–2.814	0.922	0.730	0.202–2.645	0.632	0.763	0.210–2.769		0.681
By ASM/BMI (FNIH)	0.816	0.299–2.228	0.692	0.642	0.202–2.042	0.453	0.680	0.214–2.162		0.514
***Muscle strength***										
By handgrip (20%)	5.813	2.448–13.800	<0.001*	4.129	1.366–12.480	0.012*	3.516	1.142–10.825		0.028*
By handgrip (FNIH)	3.229	0.951–10.964	0.060	5.786	0.735–45.608	0.095	5.494	0.670–45.019		0.112
***Physical performance***										
By walking velocity (20%)	2.522	1.045–6.085	0.040*	1.484	0.523–4.209	0.458	1.365	0.486–3.832		0.554
By walking velocity (FNIH)	3.842	0.895–16.493	0.070	2.124	0.450–10.025	0.341	1.774	0.376–8.363		0.469
***In combination***										
By Sarcopenia_mass/strength_ (20%)	3.464	1.020–11.764	0.046*	1.639	0.330–8.134	0.545	1.478	0.291–7.497		0.638
By Sarcopenia_mass/strength_ (FNIH)	1.211	0.444–3.306	0.709	0.904	0.283–2.886	0.864	0.886	0.274–2.846		0.840
By Sarcopenia_mass/strength/performance_ (20%)	4.182	0.973–17.966	0.054	1.115	0.125–9.937	0.923	1.272	0.139–11.664		0.832
By Sarcopenia_mass/strength/performance_ (FNIH)	1.513	0.554–4.132	0.419	1.000	0.308–3.246	0.999	0.976	0.299–3.184		0.968
By Sarcopenia (AWGS)	0.866	0.255–2.941	0.818	0.978	0.273–3.504	0.101	0.973	0.217–2.849		0.715

Hazard ratio of 6-year mortality was evaluated with multivariate Cox analysis. ASM, appendicular skeletal muscle mass; ht^2^, height squared; (20%), Lowest quintile for each variable in each gender were used to classify sarcopenia; (FNIH), Cutoff values recommended by FNIH were used to classify sarcopenia; Sarcopenia_mass/strength_ was defined as low muscle mass with low muscle strength; Sarcopenia_mass/strength/performance_ was defined as slowness with both low muscle mass and strength. Model 1: Unadjusted; Model 2: Adjusted for age, BMI, systolic blood pressure, fasting glucose, total cholesterol, creatinine, alanine transaminase, free T4, the cumulative illness rating scale; Model 3: Adjusted for covariates in Model 2, smoking, alcohol, exercise

*P < 0.05.

We explored the proportion of subjects who fell below the lowest quintile and the FNIH-recommended cutoff values for low muscle mass, weakness, slowness, and their composites (S1 Fig). When using the lowest quintile to define sarcopenia, 23 (8.1%) men were allocated to Sarcopenia_mass/strength_ and 10 (3.5%) to Sarcopenia_mass/strength/performance_, while 14 (5.1%) women were allocated to Sarcopenia_mass/strength_ and 8 (2.9%) to Sarcopenia_mass/strength/performance_. With the FNIH criteria, a higher proportion of subjects fell below each component: in men, 92 (32.3%) had low muscle mass, 141 (49.5%) had weakness, and 148 (51.9%) had slowness; in women, 76 (27.6%) had low muscle mass, 177 (64.4%) had weakness, and 196 (71.3%) had slowness. A total of 56 (19.6%) men fulfilled the criteria for Sarcopenia_mass/strength_ and 40 (14.0%) for Sarcopenia_mass/strength/performance_, and 57 (20.7%) women met the criteria for Sarcopenia_mass/strength_ and 48 (17.5%) for Sarcopenia_mass/strength/performance_.

## Discussion

In this community-based prospective cohort study, we evaluated whether the new criteria proposed by FNIH for sarcopenia could accurately represent the increased risk of mortality and provide better predictive values in Korean sarcopenic elderly men. We also found that the cutoffs by the lowest quintile predicted mortality more accurately than those by FNIH-recommended cutoffs in older Korean adults, and the composite of parameters was a better predictor for mortality than each parameter alone. These associations, however, were only observed in men, but not in women. To our knowledge, this is one of the first prospective studies in Asia that comprehensively evaluated the new FNIH criteria in conjunction with mortality for sarcopenia.

Various definitions for sarcopenia have been proposed to date, including ASM/ht^2^ [4], ASM/weight [26], and a number of definitions suggested by authorized working groups [5, 7, 8]. However, clinical implications or prevalence of sarcopenia varied according to the methods used for defining sarcopenia [11, 27]. The FNIH definition is the most recent and evidence-based criteria, derived from 9 large community-based cohorts with a total of 26,625 subjects [12]. The usual prevalence of sarcopenia ranged from 5% to 50% by criteria that are used [27], but only 0.5% men and 1.8% women met all three criteria in the FNIH study [22]. The proportion of Sarcopenia_mass/strength_ and Sarcopenia_mass/strength/performance_ in our study population was as low as 2.9%–8.1% when the lowest quintile for each parameter was applied (S1 Fig). On the other hand, this study demonstrated better prediction of mortality using the FNIH definition of sarcopenia compared with using each parameter alone or with conventional definitions of sarcopenia including the AWGS criteria [15, 17, 28]. This stringent FNIH criteria might classify fewer elderly subjects into sarcopenia, but is expected to better differentiate older persons who are frail and at risk of mortality.

Measures of muscle mass, muscle strength, and physical performance are known to differ by race [29, 30]. It is notable that a significantly higher proportion of older Korean adults had low lean mass, weakness, and slowness than those in the FNIH population (S1 Fig). In women, for instance, the proportion of subjects with both low lean mass and weakness (Sarcopenia_mass/strength)_ by FNIH cutoffs reached up to 20%, whereas it decreased to 5.1% by lowest quintile as cutoff. To investigate the appropriate cutoff values in our population, we used cutoffs of the lowest quintile for each component as suggested by a number of working groups [5, 7, 8]. In this study, the risk for mortality was significantly higher in older Korean men when using the lowest quintile (4–9-fold) than the FNIH cutoffs (2–4-fold). Our findings suggest that the cutoffs by the lowest quintile in study subjects, which reflect inter-ethnic difference, enhance the predictability of mortality related to sarcopenia, and can be a suitable method to classify sarcopenia in specific study populations.

The association between sarcopenia and 6-year mortality was strong among men, but this finding was inconsistent among women. There are some plausible explanations for the gender difference. First, physiological muscle loss due to aging tends to start later in women than men [31]. Therefore, detrimental outcomes of muscle loss in older adults might be pronounced in men. Second, gender differences in body composition might also affect these differential associations. Previous body composition analyses have shown that fat mass is relatively higher and muscle mass is lower in women than in men [32], which is consistent with the observations in our study (fat% (total body fat/body weight): men, 24.17±5.34% and women, 33.82±4.83%; lean mass: men, 49.39±6.01 kg and women, 37.10±4.17 kg). Therefore, the absolute amount of muscle loss with aging might be greater in men than in women [31], and muscle mass changes could influence health conditions to different degrees in women and men. However, the underlying mechanisms of gender differences in the association of sarcopenia and mortality warrant further investigational research.

This study has some distinctive features. We validated the defining criteria for sarcopenia recently proposed by FNIH in association with mortality among Asian population, and we observed that a new method of calculating relative muscle mass, BMI adjusted ASM, could have better clinical implications than ASM/ht^2^. We also found that the lowest quintile cutoffs better predicted mortality than the FNIH-recommended cutoffs in our study group. On the other hand, this study has some limitations. First, this study included a limited number of subjects. Moreover, few deaths occurred during the follow-up period, especially in women. The sample size and event rates were not sufficient to achieve statistical power at a level of 0.8. Therefore, we could not conclude whether the lack of significant associations in women was because of the biologic difference between men and women or because of the small numbers of female subjects and of deaths in women. However, several previous studies have reported similar results to those of our study [28]. In conclusion, a prospective longitudinal cohort study that includes large numbers of older subjects is warranted to elucidate further the clinical implication of sarcopenia in women, especially regarding mortality. Second, the longitudinal evaluation of functional performance measures might have been confounded by survival bias. The cohort in this study included older people, and subjects who had developed functional impairment (*i*.*e*., who died during follow-up) might easily have been censored in the 5-year follow-up evaluation, whereas relatively healthy subjects were able to participate in the follow-up study. This bias might distort or underestimate the effect of sarcopenia on functional deterioration. Third, because of the observational nature of the study, we could not evaluate the causal relationship or exclude possible confounding effects.

This study demonstrated that the new FNIH definition for sarcopenia, which consists of low lean mass, weakness, and slowness, predicted 6-year mortality in older Korean men. Assessment of sarcopenia in older people should be considered to assess the risk of mortality and their functional performances, while appropriate cutoff values for sarcopenia in different ethnicities should be further investigated.

## Supporting Information

S1 Table The cutoff points of different sarcopenia definitions. (DOCX)

S2 Table Baseline functional performance measures by sarcopenic status. (DOCX)

S3 Table Changes in functional performance measures by sarcopenia (Sarcopenia_mass/strength_ (20%)). (DOCX)

S4 Table Six-year mortality by sarcopenia parameters—subgroup analysis by age. (DOCX)

S1 Fig The Prevalence of Sarcopenia by the Lowest Quintile and FNIH-recommended Cutoff Values. (DOCX)
