# Supplementary material for: Predictive Values of the New Sarcopenia Index by the Foundation for the National Institutes of Health Sarcopenia Project for Mortality among Older Korean Adults
Source: PLoS One. 2016 Nov 10;11(11):e0166344. doi: 10.1371/journal.pone.0166344 (PMC5104471; doi:10.1371/journal.pone.0166344)
Supplement: S1 Table — (DOCX) [file pone.0166344.s001.docx]

**S1 Table. The cutoff points of different sarcopenia definitions**

|  | Men | | | Women | | |
| --- | --- | --- | --- | --- | --- | --- |
|  | Lowest 20% | FNIH | AWGS | Lowest 20% | FNIH | AWGS |
| ASM/ht^2^ | 6.69 |  | 7.0 | 5.42 |  | 5.4 |
| ASM/BMI | 0.753 | 0.789 |  | 0.496 | 0.512 |  |
| Handgrip strength (kg) | 18.1 | 26 | 26 | 9.1 | 16 | 18 |
| Walking velocity (m/s) | 0.50 | 0.80 | 0.80 | 0.50 | 0.80 | 0.80 |

Lowest quintile for each variable in each gender and cutoff values recommended by FNIH are presented.

ASM, appendicular skeletal muscle mass.
