# Supplementary material for: Predictive Values of the New Sarcopenia Index by the Foundation for the National Institutes of Health Sarcopenia Project for Mortality among Older Korean Adults
Source: PLoS One. 2016 Nov 10;11(11):e0166344. doi: 10.1371/journal.pone.0166344 (PMC5104471; doi:10.1371/journal.pone.0166344)
Supplement: S2 Table — (DOCX) [file pone.0166344.s002.docx]

**S2 Table. Baseline functional performance measures by sarcopenic status**

| Sarcopenia_mass/strength_ (20%) | | | | | | | | | |
| --- | --- | --- | --- | --- | --- | --- | --- | --- | --- |
|  | All | | | Men | | | Women | | |
|  | No Sarcopenia | Sarcopenia | *P* | No Sarcopenia | Sarcopenia | *P* | No Sarcopenia | Sarcopenia | *P* |
| ADL | 7.08 ± 0.47 | 7.30 ± 0.94 | 0.173 | 7.03 ± 0.16 | 7.26 ± 1.05 | 0.299 | 7.14 ± 0.64 | 7.36 ± 0.75 | 0.214 |
| IADL | 12.09 ± 3.39 | 15.65 ± 5.35 | <0.001 | 12.95 ± 3.70 | 15.87 ± 4.59 | <0.001 | 11.24 ± 2.81 | 15.29 ± 6.58 | 0.039 |
| SPPB | 7.50 ± 1.12 | 6.57 ± 1.52 | 0.001 | 7.69 ± 1.03 | 6.91 ± 1.47 | 0.021 | 7.31 ± 1.23 | 6.00 ± 1.47 | <0.001 |
| Sarcopenia_mass/strength/performance_ (20%) | | | | | | | | | |
|  | All | | | Men | | | Women | | |
|  | No Sarcopenia | Sarcopenia | *P* | No Sarcopenia | Sarcopenia | *P* | No Sarcopenia | Sarcopenia | *P* |
| ADL | 7.08 ± 0.47 | 7.30 ± 1.25 | 0.174 | 7.03 ± 0.16 | 7.60 ± 1.58 | 0.279 | 7.14 ± 0.65 | 7.38 ± 0.74 | 0.314 |
| IADL | 12.14 ± 3.40 | 18.00 ± 6.09 | 0.001 | 13.00 ± 3.69 | 18.20 ± 5.05 | <0.001 | 11.25 ± 2.82 | 17.75 ± 7.56 | 0.045 |
| SPPB | 7.48 ± 1.14 | 6.00 ± 1.78 | 0.003 | 7.67 ± 1.03 | 6.40 ± 1.78 | 0.051 | 7.29 ± 1.22 | 5.50 ± 1.77 | 0.024 |
