# Supplementary material for: Predictive Values of the New Sarcopenia Index by the Foundation for the National Institutes of Health Sarcopenia Project for Mortality among Older Korean Adults
Source: PLoS One. 2016 Nov 10;11(11):e0166344. doi: 10.1371/journal.pone.0166344 (PMC5104471; doi:10.1371/journal.pone.0166344)
Supplement: S3 Table — (DOCX) [file pone.0166344.s003.docx]

**S3 Table. Changes in functional performance measures by sarcopenia (Sarcopenia_mass/strength_ (20%))**

|  | No Sarcopenia | Sarcopenia | *P* |
| --- | --- | --- | --- |
| ADL (n=375) | 0.025 ± 0.54 | 0.27 ± 0.90 | 0.387 |
| IADL (n=375) | 2.04 ± 1.23 | 1.10 ± 3.51 | 0.485 |
| SPPB (n=191) | 2.75 ± 1.95 | 1.33 ± 3.50 | 0.091 |
