# Supplementary material for: Predictive Values of the New Sarcopenia Index by the Foundation for the National Institutes of Health Sarcopenia Project for Mortality among Older Korean Adults
Source: PLoS One. 2016 Nov 10;11(11):e0166344. doi: 10.1371/journal.pone.0166344 (PMC5104471; doi:10.1371/journal.pone.0166344)
Supplement: S4 Table — (DOCX) [file pone.0166344.s004.docx]

**S4 Table. Six-year mortality by sarcopenia parameters – subgroup analysis by age**

|  | Alive | Death | *P* |
| --- | --- | --- | --- |
| Men: Sarcopenia_mass/strength_ (20%) | | | |
| Age: 65-74 |  |  | <0.001 |
| No Sarcopenia | 172 (99.4%) | 11 (78.6%) |  |
| Sarcopenia | 1 (0.6%) | 3 (21.4%) |  |
| Age: 75- |  |  | 0.024 |
| No Sarcopenia | 61 (85.9%) | 17 (65.4%) |  |
| Sarcopenia | 10 (14.1%) | 9 (34.6%) |  |
| Men: Sarcopenia_mass/strength/performance_ (20%) | | | |
| Age: 65-74 |  |  | <0.001 |
| No Sarcopenia | 173 (100.0%) | 13 (92.9%) |  |
| Sarcopenia | 0 (0.0%) | 1 (7.1%) |  |
| Age: 75- |  |  | <0.001 |
| No Sarcopenia | 70 (98.6%) | 18 (69.2%) |  |
| Sarcopenia | 1 (1.4%) | 8 (30.8%) |  |
| Women: Sarcopenia_mass/strength_ (20%) | | | |
| Age: 65-74 |  |  | 0.070 |
| No Sarcopenia | 181 (98.4%) | 9 (90.0%) |  |
| Sarcopenia | 3 (1.6%) | 1 (10.0%) |  |
| Age: 75- |  |  | 0.527 |
| No Sarcopenia | 62 (88.6%) | 9 (81.8%) |  |
| Sarcopenia | 8 (11.4%) | 2 (18.2%) |  |
| Women: Sarcopenia_mass/strength/performance_ (20%) | | | |
| Age: 65-74 |  |  | 0.004 |
| No Sarcopenia | 183 (99.5%) | 9 (90.0%) |  |
| Sarcopenia | 1 (0.5%) | 1 (10.0%) |  |
| Age: 75- |  |  | 0.819 |
| No Sarcopenia | 65 (92.9%) | 10 (90.9%) |  |
| Sarcopenia | 5 (7.1%) | 1 (9.1%) |  |
