# Supplementary material for: Predictive Values of the New Sarcopenia Index by the Foundation for the National Institutes of Health Sarcopenia Project for Mortality among Older Korean Adults
Source: PLoS One. 2016 Nov 10;11(11):e0166344. doi: 10.1371/journal.pone.0166344 (PMC5104471; doi:10.1371/journal.pone.0166344)
Supplement: S1 Fig — (DOCX) [file pone.0166344.s005.docx]

**S1 Figure. The prevalence of sarcopenia by the lowest quintile and by the FNIH-recommended cutoff values**


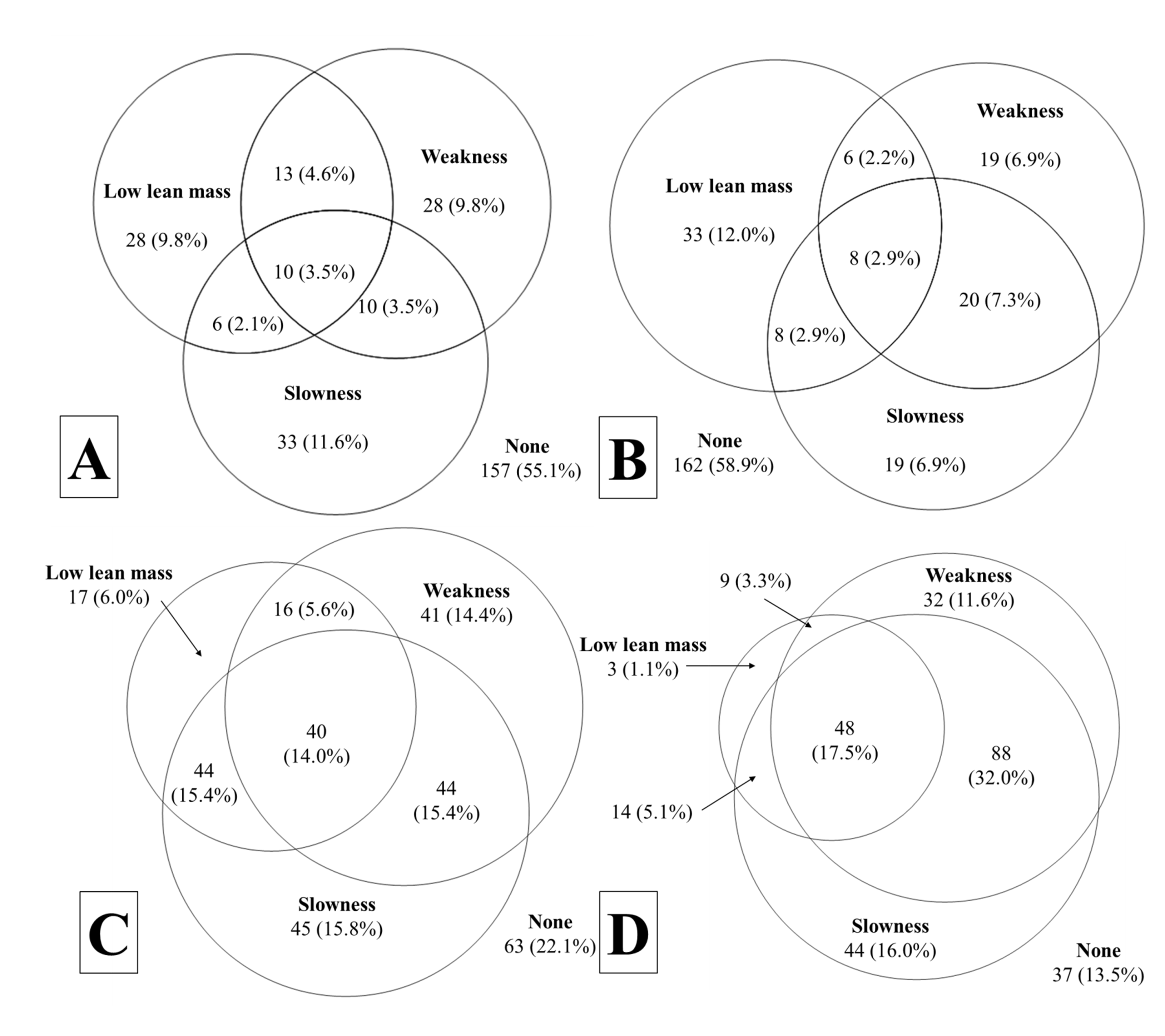


Numbers of subjects meeting each criterion for sarcopenia by the lowest quintile (20%) or FNIH-recommended cutoff values (FNIH) are shown in Venn diagram (A, men (20%); B, women (20%); C, men (FNIH); D, women (FNIH)). The area of each circle is proportional to the number of subjects. Numbers in overlapped region represents subjects meeting both or three criteria.
